# Surgical Anatomy of the Gastrointestinal Tract in Cats

**DOI:** 10.3390/ani13162670

**Published:** 2023-08-19

**Authors:** Vasileia Angelou, Aliki Fiska, Anastasia Tsingotjidou, Michael Patsikas, Lysimachos G. Papazoglou

**Affiliations:** 1 Unit of Surgery and Obstetrics, Companion Animal Clinic, School of Veterinary Medicine, Faculty of Health Sciences, Aristotle University of Thessaloniki, 54627 Thessaloniki, Greece; vasso_1991@hotmail.com (V.A.); makdvm@vet.auth.gr (L.G.P.); 2 Department of Anatomy, Medical School, Democritus University of Thrace, 68100 Alexandroupolis, Greece; afiska@med.duth.gr; 3 Laboratory of Anatomy, Histology and Embryology, School of Veterinary Medicine, Faculty of Health Sciences, Aristotle University of Thessaloniki, 54627 Thessaloniki, Greece; astsing@vet.auth.gr; 4 Laboratory of Diagnostic Imaging, Department of Clinical Studies, School of Veterinary Medicine, Faculty of Health Sciences, Aristotle University of Thessaloniki, 54627 Thessaloniki, Greece

**Keywords:** abdomen, anatomy, feline, gastrointestinal surgery

## Abstract

**Simple Summary:**

The surgical anatomy of the feline gastrointestinal tract has not been studied thoroughly, and there are only a few anatomical studies in the literature. Surgical procedures carried out on the gastrointestinal tract of cats are quite frequent, and their deep knowledge of surgical anatomy can help veterinary surgeons to better understand the anatomy of the gastrointestinal tract and plan and perform the main surgical procedures used in this region. The purpose of the present study is to describe the anatomy of the feline gastrointestinal tract and mention the basic surgical procedures that are performed in each region, as well as some surgical tips for veterinary surgeons.

**Abstract:**

In cats, the gastrointestinal tract is one of the regions in which surgical procedures are most frequently performed by veterinary surgeons; therefore, knowledge of the surgical anatomy of the feline gastrointestinal tract is of high importance. The main surgical procedures performed include gastrotomy, gastrectomy, enterotomy, and enterectomy, as well as procedures in the liver and pancreas. There are also anatomical differences between dogs and cats, increasing the need for deep knowledge of the anatomy treated in the different surgical approaches. The aim of the present review is to describe in detail the anatomy of the gastrointestinal tract in cats highlighting the anatomical regions of significant importance in different surgical procedures.

## 1. Introduction

Feline surgery has rapidly developed in the last few decades, and there are surgical operating conditions unique to this species [1]. The anatomy of the cat also has differences compared to that of the dog, making knowledge of surgical anatomy indispensable when performing surgical approaches to treat different conditions. 

The surgical anatomy of the gastrointestinal tract of the cat has not been extensively studied, and most such studies that focused on anatomy are not up-to-date [2,3]. Deep knowledge of the surgical anatomy of the gastrointestinal tract is a useful tool for surgeons and practitioners because the surgical procedures performed in this anatomical region are quite common. The most frequently performed surgical procedures include gastrotomy and enterotomy for foreign body removal, gastrectomy, and enterectomy, colectomy in cats with idiopathic megacolon, and surgical procedures in the pancreas and liver [4,5,6,7,8,9,10]. This is the reason that the detailed description of surgical anatomy and the surgical procedures that are performed in each of the anatomical regions of the gastrointestinal tract can contribute to better pre-surgical planning of the procedures by the veterinarian. Therefore, proper education regarding surgical anatomy is important because both surgical trauma and different surgical diseases can alter normal anatomy [11]. 

The aim of the present review is to describe the anatomy of the gastrointestinal tract of the abdomen in cats in combination with the surgical procedures that are performed in each region, highlighting the points of surgical interest. All of the images of the present study were obtained from cases seen by the authors. In all cases, the owner’s consent was obtained. 

## 2. Ventral Abdominal Wall

**Figure 1:**
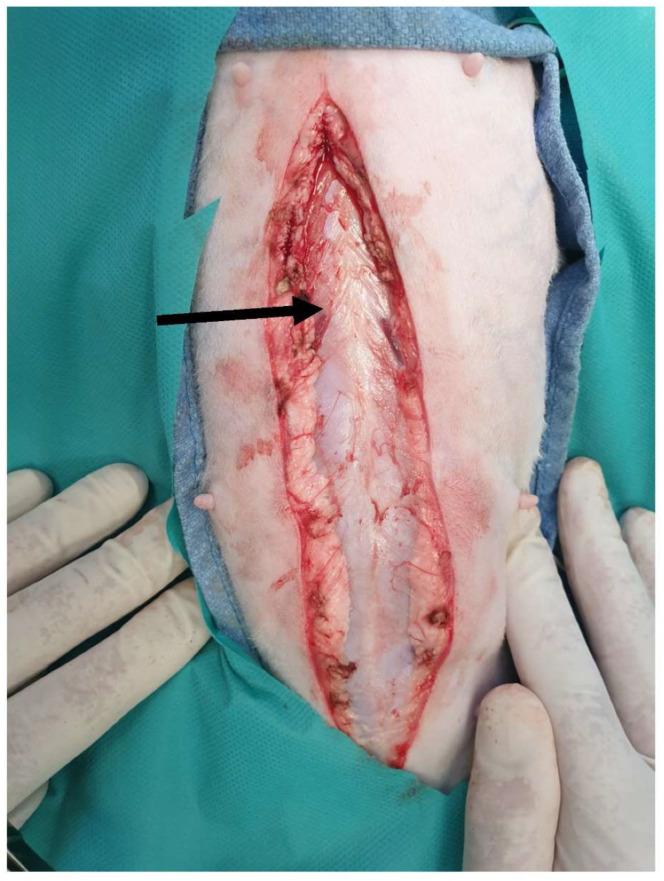
The linea alba is identified (arrow) following midline incision in the skin and subcutaneous tissue.

**Figure 2:**
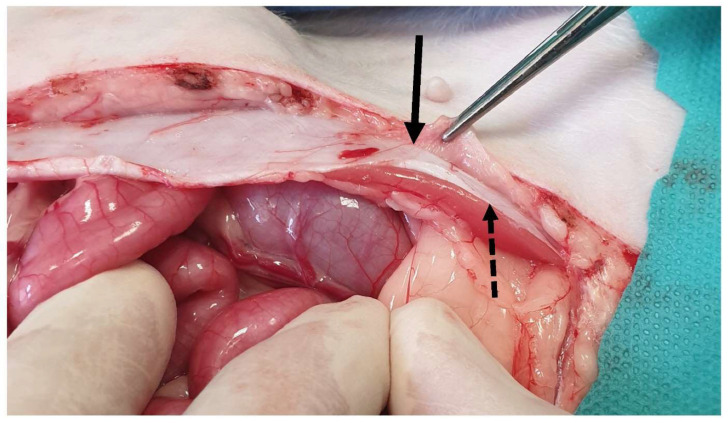
Following midline incision in the ventral abdominal wall, the rectus abdominis muscle (dotted arrow) and the external leaf of the rectus sheath (black arrow) are exposed.

**Figure 3:**
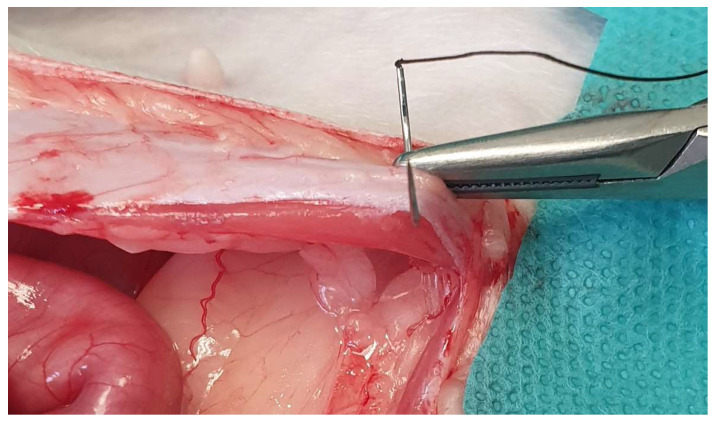
For celiotomy closure, only the external leaf of the rectus sheath should be included in the suture line.

At the ventral midline, the linea alba, which is a fibrous band formed by the convergence of the tendinous aponeuroses of the external abdominal oblique, internal abdominal oblique, and transversus abdominis muscles can be observed; in cats, the linea alba is as wider as 4 mm, semitransparent, and easily identified compared to those of dogs [12,13,14] (Figure 1). The rectus abdominis muscle is covered by two aponeuroses of the other abdominal muscles: the external and internal leaves of the rectus sheath (Figure 2). In the caudal third of the abdominal wall, the internal leaf does not exist. In a cadaveric study, the thickness of the linea alba ranged from 0.6 to 1.98 mm (1.13 ± 0.40 mm) [15]. It has been found that the location of the aponeurosis of the internal abdominal oblique differs as the pre-umbilical side is both deeply and superficially located relative to the rectus abdominis, and the umbilical and post-umbilical sides are only superficially located [14]. It has been reported that the post-umbilical region is weaker and thinner [15]. Midline celiotomy or laparotomy is the standard incision method used to access the abdominal cavity. Celiotomy is the most common procedure performed on dogs and cats. The extent of celiotomy depends on the abdominal organ being exposed, but for abdominal exploration, celiotomy should extend from the xiphoid process to the pubic symphysis [13]. For celiotomy closure, only the external leaf of the rectus sheath should be included in the suture line in cats, as it was proven to be the holding layer of the incision [15] (Figure 3). In cats, a pre-pubic tendon does not exist, but attachments of the aponeurosis of the external abdominal oblique muscle to the tendon of the pectineus support the area, which is considered to be as strong as the pre-pubic tendon in dogs [16]. 

## 3. Digestive Tract 

### 3.1. Stomach 

The stomach is a C-shaped organ, the largest organ in terms of size in the gastrointestinal tract, and located between the esophagus and the duodenum [17,18]. It communicates with the esophagus via the cranial and left sides of the inlet of the stomach, which is known as cardia [17]. The distal and narrowest part of the stomach is connected to the duodenum via a muscular sphincter in the cranial part of the duodenum, which is known as pylorus, with its lumen being called ostium pyloricum [19]. The largest part of the stomach lies on the left cranial side of the abdomen, with the cranial surface being connected to the diaphragm and the visceral surface being connected to the liver [17]. The cardia of the stomach is not directly connected to the diaphragm because the distal, which is a small part of the esophagus, is inserted into the abdomen in communication with the stomach [20]. 

The feline stomach has a capacity of approximately 300–350 mL [21]. It is also known that in dogs and cats, the position of the stomach changes depending on the filling and the size of the remaining viscera [21]. More specifically, there are studies reporting the varying changes in the topography of liver lobes, kidneys, small intestine, and colon depending on the stomach filling [22,23,24]. 

**Figure 4:**
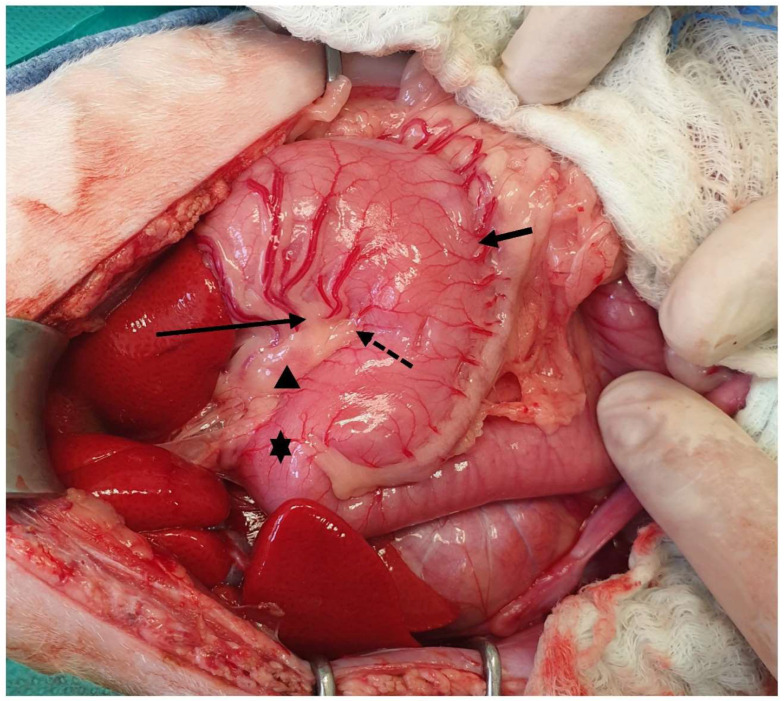
The lesser curvature (arrow), the angular notch (dotted arrow), the greater curvature (short arrow), the pylorus (asterisk), and a gastric lymph node (arrowhead) are visualized. The head is located on the left side of the image.

**Figure 5:**
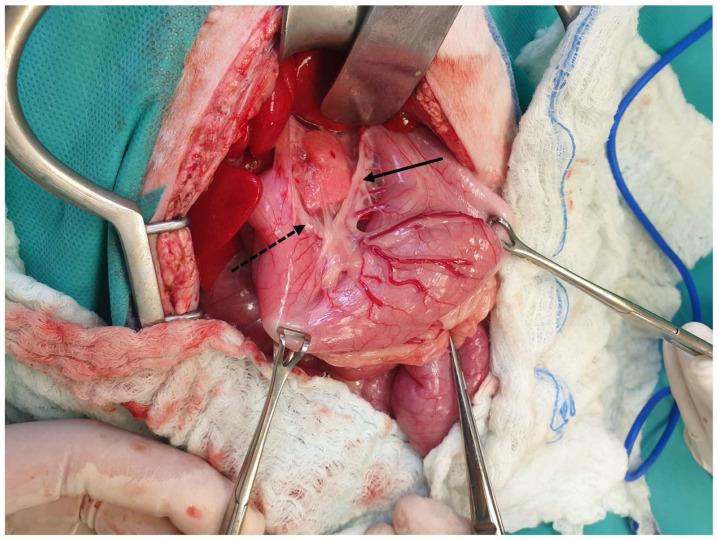
The left gastric artery (solid arrow) and the right gastric artery (dotted arrow) are exposed following the caudal traction of the stomach using a pair of Babcock forceps. The head is located in the upper part of the image.

**Figure 6:**
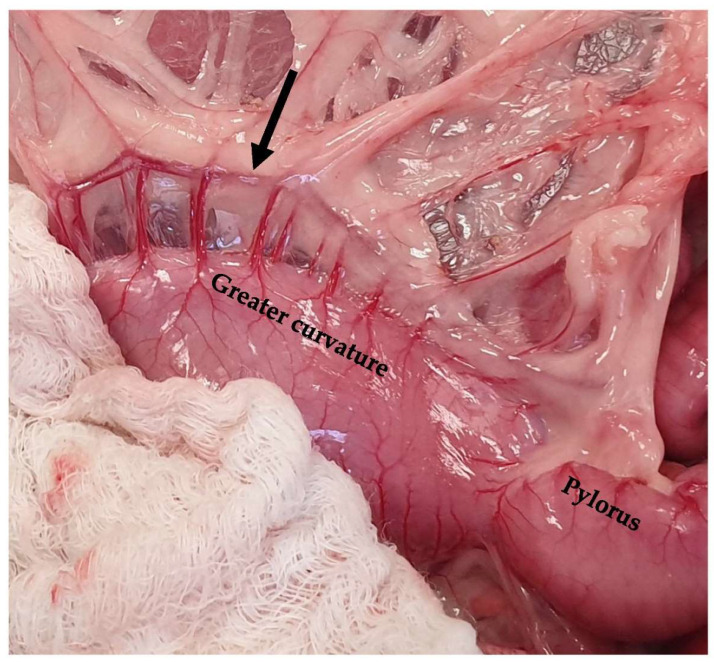
The gastroepiploic artery is visualized (arrow) following the ventral traction of the omentum. The head is located on the left side of the image.

The stomach in cats, as in dogs, consists of two curvatures, i.e., the lesser and the greater curvature, which distinguish the stomach into two sites, i.e., a concave and a convex site. The lesser curvature, which forms a 50–70-degree angle, is the concave side and extended in a cranial position from the cardia to the pylorus [17,19]. The lesser curvature has an angular notch between the body and the pyloric part of the stomach [20]. This angular notch is deeper in cats than in dogs, making the process of gastroscopy more difficult [25]. The lesser curvature is supplied by the left gastric artery, which is a direct branch of the celiac artery, and it has anastomosis with the right gastric artery, with the satellite vein being derived from the gastrosplenic vein [19,20]. The lymph nodes of the stomach are located on the lesser curvature within the lesser omentum, close to either the cardia or the pylorus [21,26]. However, gastric lymphatic drainage occurs through the gastric, splenic, and hepatic lymph nodes [27]. The greater curvature is the convex part of the stomach, which also extends from the cardia to the pylorus in contact with the floor of the abdomen and the spleen [20,25]. The greater curvature is supplied by the left gastroepiploic artery, i.e., a branch provided by the splenic artery, which connects to the right gastroepiploic, i.e., a branch of the gastroduodenal artery, as well as by the short gastric arteries and veins and the tributaries of the splenic artery and vein [19,20]. The left gastroepiploic veins are branches of the splenic vein, and the right gastroepiploic veins are branches of the gastroduodenal vein [19,20]. All gastric veins are tributaries of the portal vein [20] (Figure 4, Figure 5 and Figure 6). 

As mentioned above, the main parts of the stomach in cats are the cardia, the fundus, the body of the stomach, and the pylorus [25]. 

**Figure 7:**
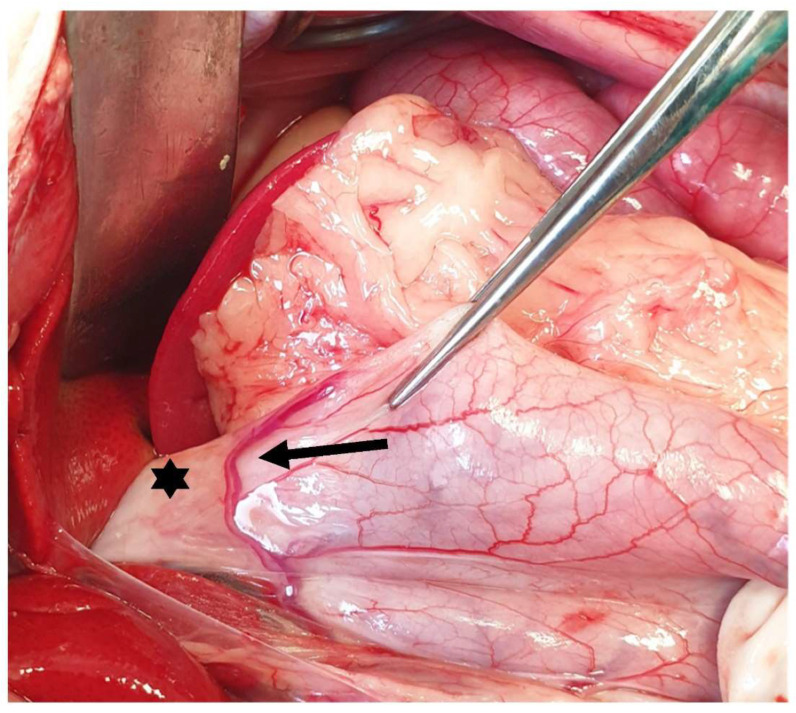
The gastric cardia (asterisk) is exposed following caudal traction of the stomach. The esophageal branch of the left gastric artery is evident (arrow). The head is located on the left side of the image.

The cardia, which is the cranial part of the stomach that is connected to the esophagus, lies on the left side of the midline [25] (Figure 7). The esophagogastric junction is an anatomical region that is clinically important in cases of hiatal hernias [28]. This is the region in which the esophagus passes caudally to the diaphragm and is inserted into the stomach, with a part of the esophagus being intra-abdominal in nature [28]. The phrenoesophageal membrane, with its ascending and descending limb, is the ligament that connects the esophagus to the diaphragm, with the descending limb being connected to the stomach just below the gastroesophageal junction [29]. However, Voutsinou et al. reported the absence of abdominal esophagus in nine domestic shorthaired cats and one domestic longhaired cat, with the zone of mucosa transition being detected between the bifurcation of the diaphragm and the phrenoesophageal membrane [30]. The gastroesophageal junction is also surgically important during the localization of the gastro azygos shunt, which usually runs adjacent to it [31]. The blood supply of the cardia is provided by the left gastric artery and the satellite vein [19,20,27]. The main surgical techniques in this anatomical region include phrenoplasty and esophagopexy performed for the treatment of hiatal hernia [21,27,32,33]. It is also important during gastrectomy to avoid cardia [34]. 

The fundus is the part of the stomach resembling a blind sac and extending to the left and dorsal to the cardia [17,20]. The mean fundus wall thickness in cats measured via ultrasound has been found to be 1.7–4.38 mm [35,36]. It is supplied by the left gastric artery and vein [19,20]. The main surgical techniques used in this anatomical region include Nissen fundoplication, left-sided gastropexy, gastrotomy, and gastrectomy. More specifically, Nissen fundoplication and modified Nissen fundoplication are surgical techniques described for the surgical treatment of hiatal hernia [32,37,38,39]. A left-sided fundal gastropexy is also performed alone or in combination with the previously described techniques for the surgical treatment of hiatal hernia in cats [33,37,40]. It has been reported that the combination of phrenoplasty, esophagopexy, and left-sided incisional gastropexy is the procedure of choice for the surgical treatment of hiatal hernia in cats [37]. Left-sided gastropexy has also been described in cases of gastroesophageal intussusception in cats [41]. Gastrotomy can be performed in the fundus, but the body of the stomach is usually preferred [20]. Gastrectomy in the fundus has been performed for the resection of the gastric diverticulum in cats, which is a condition rarely described in the literature, with the fundus being the region in which the procedure typically occurs [42]. 

**Figure 8:**
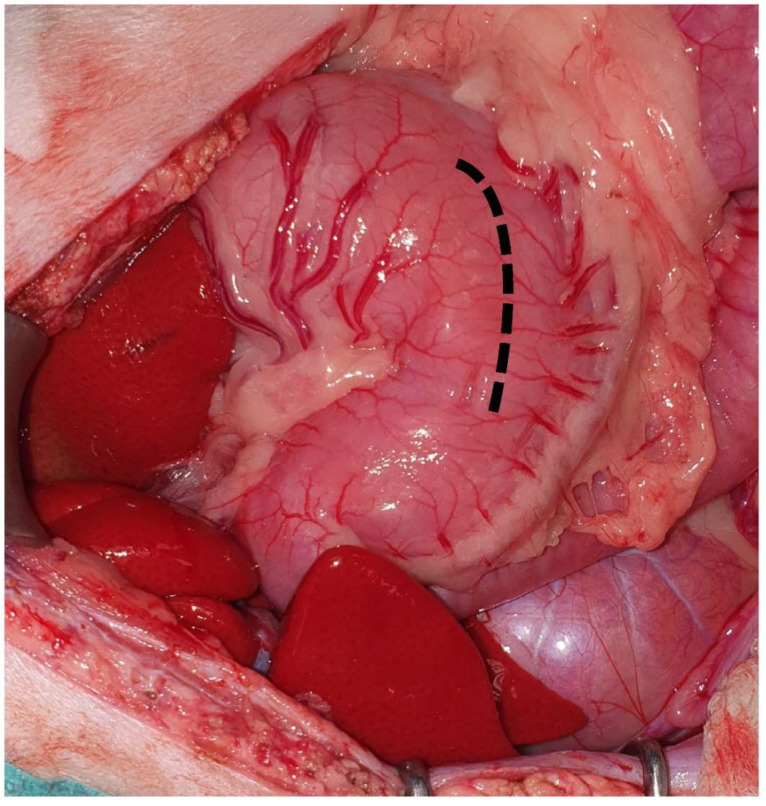
The gastrotomy is performed in a hypovascular area between the greater and lesser curvatures on the ventral surface of the stomach (dotted line). The head is located on the left side of the image.

The body is the largest part of the stomach, extending from the fundus on the left side to the pylorus on the right side [25]. The main surgical procedures performed in the body of the stomach include gastrostomy tube placement, gastrotomy, and gastrectomy. A gastrotomy is usually performed to remove foreign bodies, inspect the gastric lumen, or obtain full-thickness biopsies [43,44]. During gastrotomy, the big vessels should be avoided, and the incision is performed in a rather hypovascular area between the greater and the lesser curvature on the ventral surface of the stomach [45] (Figure 8). The incision should be performed away from the pylorus to avoid the narrowing of the gastric lumen [43]. If gastrectomy is planned, gastrotomy should be performed at a side that allows luminal inspection, avoiding interference with the gastrectomy plan [21]. Segmental gastrectomy can also be performed in the body of the stomach, with the left and right gastric and gastroepiploic vessels needing to be ligated [34]. Segmental gastrectomies are usually performed due to gastric ulceration post-NSAID administration in cats [5,46]. A laparoscopic sleeve gastrectomy has also been described in 12 cats (10 cadavers and 2 cat subjects), with the gastrectomy being performed at 4 cm over the pylorus to the lesser curvature at the point of the left gastric vessels. It is suggested that the resection should not be performed too close to the lesser curvature (<1.5 cm) due to the possibility of causing stenosis [47].

The pylorus is the distal part of the stomach, which is divided into the pyloric antrum, i.e., the wider side of the pylorus, and the pyloric canal, i.e., the narrowest part of the stomach [17,20,25]. Feline pyloric diameter ranges from 8 to 10 mm [48]. In the region in which the pylorus connects to the duodenum, there is a thickening called the pyloric sphincter [17,20]. The pylorus and the pyloric antrum are supplied by the right gastric artery, which is anastomosed to the left gastric artery, i.e., a branch of the hepatic artery, and by the satellite vein [20]. The right gastric vein is a branch of the gastroduodenal vein [19,20]. The pyloric antrum is the region in which the different gastropexy techniques are performed. In cats, a right-sided incisional and belt loop gastropexy has previously been described [49,50]. However, gastropexy carried out to treat gastric dilatation and volvulus (GDV) is not commonly performed in cats due to the different etiology compared to that of dogs [49]. Gastrocolopexy has not been described in cats, unlike in dogs, regarding the performance of GDV treatment [51,52]. Pyloric surgical procedures are rarely performed on cats. Pyloric procedures include Fredet–Ramstedt pyloromyotomy, Heineke–Mikulicz pyloroplasty, and Y-U advancement pyloroplasty, which was first described in 2022 [53,54,55,56]. During the Y-U pyloroplasty, the arms of the Y are located in the pyloric antrum [57]. The tip of the U flap should be curved to decrease the risk of necrosis and increase the vascular supply [57,58]. The pylorus is not preferred for gastrotomy incisions due to the risk of stenosis [43]. When performing a Billroth I, which is rarely described in cats, it is important to take care not to ligate the bile duct [59,60,61]. The right gastric and right gastroepiploic vessels are ligated for this procedure [57]. Billroth II has been described in two cats due to the need for the surgeon to manage extensive tension for gastric perforations [62]. During this procedure, the pyloric antrum should be resected, and, in general, it is considered to be a highly demanding procedure, especially for the inspection of the dorsal aspect of the distal extent of gastric incision [62,63]. The Roux-en-Y surgical procedure has not been described in cats. 

**Figure 9:**
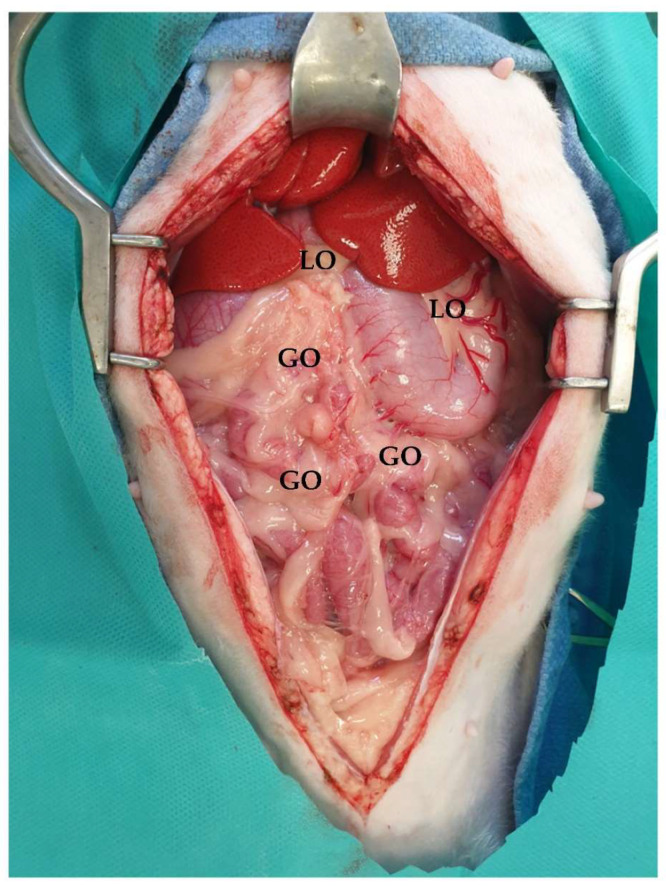
Midline celiotomy was performed, and greater (GO) and lesser omentum (LO) were evident. The head is located in the upper part of the image.

The omentum is divided into the lesser and greater omentum, which are attached to the lesser and greater curvature, respectively [25]. The lesser omentum connects the liver to the stomach and the duodenum. The lesser curvature is connected to the caudal region of the liver via the hepatogastric ligament, which is a part of the lesser omentum [20]. This ligament is transected to improve the exposure of the stomach to the pylorus during surgery, including pylorectomy and gastroduodenostomy [20,27]. The greater omentum includes the bursal, splenic, and veil portions. The bursal part of the omentum is attached to the greater curvature and continues to the urinary bladder, and it is connected to the lesser omentum to close the omental bursa [64]. The splenic part of the omentum is the gastrosplenic ligament, which contains the gastroepiploic vessels (Figure 9). 

### 3.2. Small Intestine 

It is well known that the intestine occupies the largest part of the abdomen, and its length is approximately three times the length of the trunk of the cat, i.e., about 1 to 1.5 m [65]. The small intestine, which extends from the pylorus to the ileocolic junction, is divided into three parts: the duodenum, the jejunum, and the ileum [66]. 

**Figure 10:**
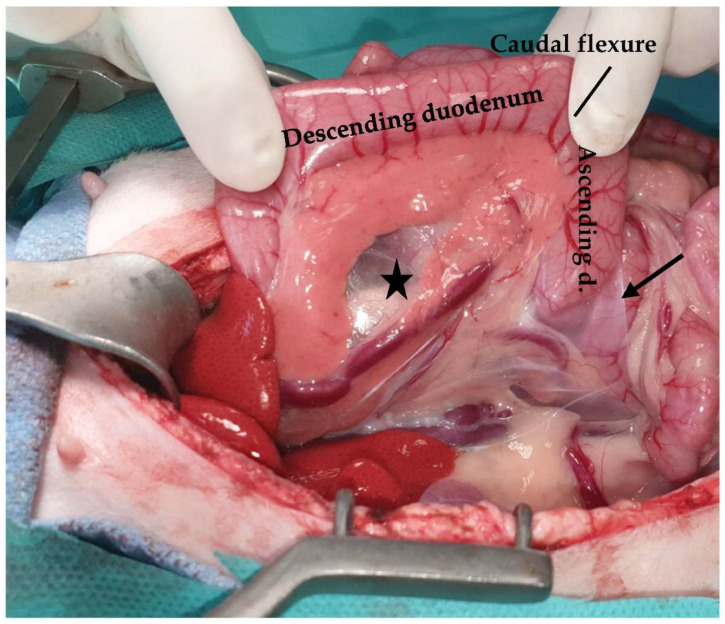
The mesoduodenum (asterisk) is retracted to the left to improve visualization of the right sublumbar region (ascending d: ascending duodenum; duodenocolic fold: arrow).

**Figure 11:**
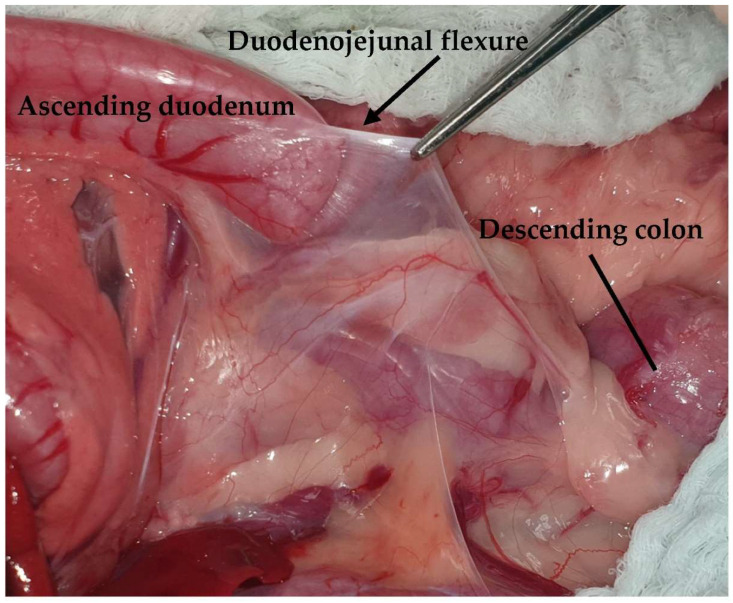
The duodenocolic fold is retracted using Debakey forceps. The head is located on the left side of the image.

The duodenum, which has a length of approximately 14–16 cm and a mean wall thickness of 2.2 mm, extends from the pylorus, creating an angle, and continues as the descending duodenum to the right side of the abdomen [67]. The descending duodenum is found in the free border of the mesoduodenum. During surgery, the mesoduodenum is retracted to the left in order to improve the visualization of the right sublumbar region, including the right kidney, adrenal gland, or right ovary. The cranial part of the duodenum creates the cranial duodenal flexure [25] (Figure 10). The descending duodenum extends near the pelvic inlet and turns, creating the caudal duodenal flexure, from right to left, continuing as the ascending duodenum. The caudal flexure of the duodenum connects the ascending and descending parts. The ascending portion runs to the left of the caudal flexure and creates the duodenojejunal flexure, which continues with the jejunum. The cranial part of the ascending duodenum is connected to the descending colon via a peritoneal fold called the duodenocolic fold, which prevents the free movement of the duodenum in the abdomen and can be transected to better expose the duodenum [66] (Figure 11). The duodenum is supplied by the cranial and caudal pancreaticoduodenal arteries, branches of the gastroduodenal artery, and by the branches of the cranial mesenteric artery [19,66]. The gastroduodenal vein and the cranial mesenteric vein drain the cranial and caudal duodenum, respectively, before terminating in the portal vein [66]. 

**Figure 12:**
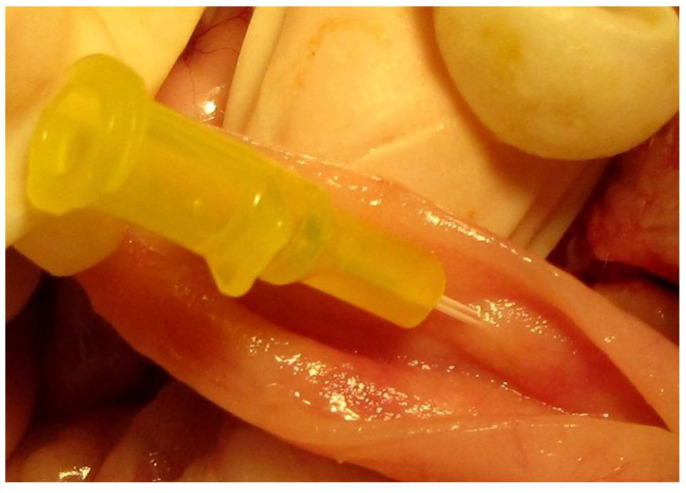
The major duodenal papilla is catheterized using a 24-gram venous catheter following a duodenotomy. The head is located on the right side of the image.

The proximal part of the duodenum contains the major duodenal papilla, which is located about 3 cm caudal to the pylorus. The major duodenal papilla receives the common bile duct and the pancreatic duct, which are conjoined before the entry to the papilla, representing an anatomical difference to dogs, in which the common bile duct enters at the major duodenal papilla, but not conjoined with the pancreatic duct, and the accessory pancreatic duct enters at the level of minor duodenal papilla [68]. The proximal duodenum is of major surgical significance, as the surgical removal of a lesion located there may necessitate a duodenectomy, pylorectomy, and cholecystoenterostomy to bypass the common bile duct that is transected during the procedure [69]. In addition, during Billroth I, care must be taken not to transect the common bile duct [61,66]. In 20% of cats, there is an accessory pancreatic duct at the minor duodenal papilla [70]. Coeuriot et al. also found that the size of the duodenal papilla is not affected by age, weight, or sex, but the type of food provided may be a factor changing the length of the papilla, as cats that eat both dry and wet food were found to have a longer papilla [70]. The major duodenal papilla is catheterized during either the evaluation of the patency of the extrahepatic biliary system or the placement of choledochal stenting, which has also been described in cats [66,71,72] (Figure 12). During this procedure, care must be taken not to catheterize the pancreatic duct in cats due to the anatomical connection between the pancreatic and the common bile duct [66]. 

**Figure 13:**
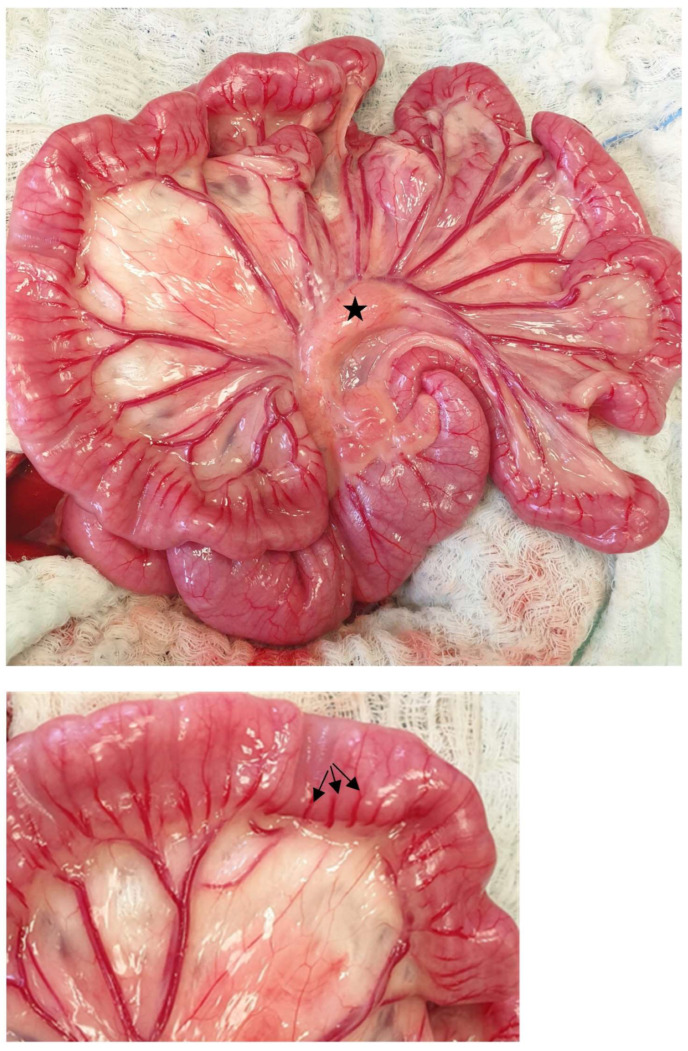
The jejunum is exposed. The branches of the cranial mesenteric artery and a jejunal lymph node are evident (asterisk). The vasa recta (arrows) are visualized in a close-up image. The head is located on the left side of the image.

**Figure 14:**
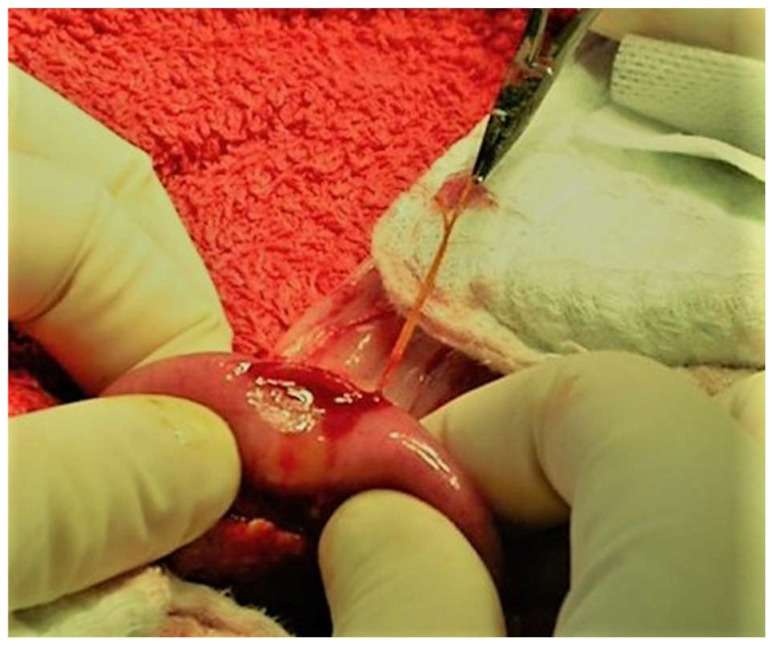
An enterotomy is performed along the antimesenteric region of the jejunum for the removal of a linear foreign body grasped by a hemostat. The head is located on the left side of the image.

The jejunum is the largest part of the small intestine, having a mean length of 130.6 ± 23.4 cm and extending from the duodenojejunal flexure to the ileocolic orifice [66,73] (Figure 13). The wall thickness, as measured via ultrasound, is reported to be 2.22 ± 0.18 mm, with the thickest layer of jejunum being the mucosa [67]. The jejunum is supplied by the branches of the cranial mesenteric artery. More specifically, in cats, it has been found that there are 5–15 jejunal branches, with each branch being further divided into 1–6 smaller branches that terminate in arcades, creating a marginal artery [73]. Vasa recta are capillaries that come from the marginal artery and further divided into vasa brevia and vasa longa, which are short and long branches, respectively [73]. The vasa brevia supply the mesenteric side of the jejunum, and the vasa longa enter beneath the serosa and supply the antimesenteric part of the jejunum [73]. Grandis et al. hypothesized that this rich network in the feline jejunum may affect the blood supply, resulting in less ischemic phenomena and less anastomotic dehiscence than in other species [73]. The venous drainage of the jejunum occurs through the portal vein via the cranial mesenteric vein [66]. Schreurs et al. reported that the jejunal lymph nodes are most frequently found in the abdomen via ultrasound examination, having a size compatible with those reported in the literature [26]. Among the surgical procedures performed in this region, enterotomy is most frequently performed for foreign body removal or biopsy, with the jejunum being the segment of the small intestine in which the foreign bodies are usually located in cats [4]. Enterotomy is performed in the antimesenteric side of the intestine to avoid damaging the blood supply [20,66] (Figure 14). The mesenteric side is not easily visualized, as it is covered by the mesentery and mesenteric fat. However, a recent report on a dog described the primary closure of a mesenteric duodenal perforation to avoid duodenectomy and more extensive surgery, including cholecystoenterostomy [74]. The submucosa is the layer of the intestine that should be included while suturing the small intestine, and it is considered to be the strongest part of the intestinal wall [66,75]. Linear foreign bodies represent one of the most common indications of enterotomy in cats [76]. The linear foreign body involved in the continued intestinal peristaltic activity may be embedded in the mesenteric region, resulting in vascular compromise, ischemic necrosis, perforations along the mesenteric side, and subsequent peritonitis. Examination of the mesenteric side though not easily performed is required for early diagnosis of possible perforations. Multiple enterotomies along the antimesenteric region are needed for the removal of the whole linear foreign body. Enterectomy is performed in cats to enable mass removal, the management of intestinal perforations, non-reducible intussusceptions, necrosis, and mesenteric volvulus. Following enterectomy, anastomosis can be either end-to-end or side-to-side in nature [66]. However, in a recent retrospective study, stenosis was reported to be a long-term complication following side-to-side anastomosis, meaning that end-to-end anastomosis is considered to be the method of choice for enterectomy in both dogs and cats [7]. Jejunum is also a region in which intussusception may occur [77,78]. Enteroplication is the procedure described in order to avoid recurrence of the intussusception. However, the use of enteroplication in cats remains controversial [78]. A jejunostomy tube can also be inserted into the proximal part of the jejunum. Finally, a loop of jejunum can be used as a serosal patch in cases of intestinal anastomosis that require support and healing augmentation of the anastomosis side [79]. 

**Figure 15:**
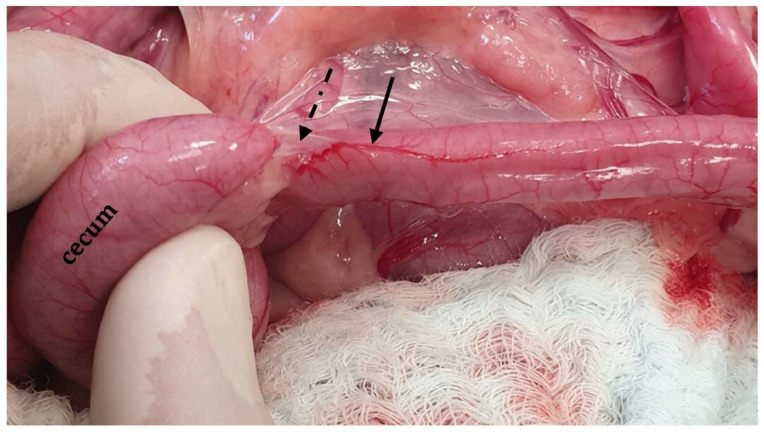
The ileum is exposed via traction of the jejunum in a ventral direction. The ileum is identified based on the antimesenteric ileal blood supply (black arrow) and the ileocecal fold (dotted arrow).

The ileum is the terminal and shortest portion of the small intestine, which is easily identified by the antimesenteric ileal blood supply and the ileocecal fold [19,20,65] (Figure 15). The length of the feline ileum has been reported to be 2.49 cm, and the wall thickness of the ileum at the level of the fold as measured via ultrasonographic examination was 3 ± 0.28 mm [67,80]. That part of the ileum is the thickest of the small intestine segments [67]. The ileum is supplied by the cecal artery in the mesenteric portion and the ileal branches of the ileocecal artery on the antimesenteric side, with no venous branches found on the two sides [19,20]. The ileum in cats is drained by the jejunal and colic lymph nodes, which may be identified via ultrasonographic examination [20,26]. The connection between the ileum and the colon occurs through the ileocolic valve, i.e., an orifice formed by the mucosa and transverse muscle layer of the ileum into the colon [20,65]. The ileocolic valve is an anatomic region of surgical importance because it avoids reflux of colonic content to the ileum [66,81]. The ileocolic valve may be resected during colectomy in cats, even though it has been suggested to be preserved due to post-operative complications, including liquid feces [8,82]. According to a systematic review, there is only one study that reported bacterial overgrowth after ileocolic valve resection [83,84]. The resection of the ileocolic valve during colectomy may be preferred because it is less demanding as long as the tension in the anastomotic side is reduced [8]. However, Grossman et al. suggest preservation of the valve due to better outcomes [8]. In the region of the ileum, enterotomy or enterectomy can also be performed. The ileocolic junction has been described as the most frequent side of gastrointestinal neoplasms in cats [85]. It is a region in which intussusception can also occur [86]. Finally, the ileum has been used as a graft for an obstructed ureter in a cat with good results [87]. 

### 3.3. Large Intestine 

The abdominal part of the large intestine is divided into two parts: the cecum and colon, which are further subdivided into ascending, transverse, and descending colon [25]. The mean feline large intestinal length was reported to be 326 ± 51 mm, representing 20.9 ± 20% of the total intestinal length [88]. The cecum is a small blind-ended pouch, which is 2–3 cm long, that represents a diverticulum connected to the remaining colon [20,89]. The cecum is located medial to the descending duodenum and caudolateral to the ileocolic junction [90,91]. The cecum in cats, unlike dogs, is a small comma or bud-shaped anatomical region with a cecocolic orifice, and it is adjacent to the ileocolic orifice [2,20,89,92]. The ileum enters the cecum from the left side, as fixed by the ileoceacalis ligament [2]. The cecum is supplied by the ileocolic artery, the branch of the cranial mesenteric artery, and the cecal artery branch of the ileocolic artery, which runs along the dorsal part of the cecum with the veins accompanied by the corresponding arteries draining to the portal vein [20,89]. At the ileocecal region, the ileocolic artery is subdivided into two vessels, with one vessel supplying the ventral part of the cecum and one smaller vessel supplying the dorsal part and a part of the ileum [2]. These branches are dissected and ligated via typhlectomy [93]. Cecal lymph nodes in cats are located in the ileoceacalis ligament, and the lymph is also drained by the colic and caudal mesenteric lymph nodes [20,92]. The main surgical procedure described for this anatomical region is typhlectomy, even though it is rarely performed. Typhlectomy is performed for the management of cecal inversion [94]. Typhlectomy can be performed using staples, which are supplied using either a thoracoabdominal (TA) or gastrointestinal anastomosis (GIA) stapling device [94]. The typhlectomy can also be performed through a colotomy when cecum inversion does not permit direct resection [95]. 

**Figure 16:**
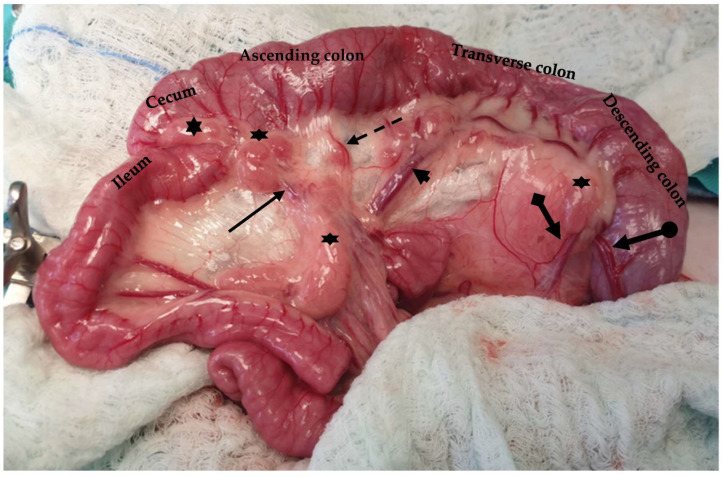
The colon is subdivided into ascending, transverse, and descending sections. Major blood supply (black arrow: ileocolic artery; dotted arrow: right colic artery; arrowhead: middle colic artery; pointed arrow: left colic artery; circle pointed arrow: cranial rectal artery) and mesenteric lymph nodes (asterisks) are visualized.

**Figure 17:**
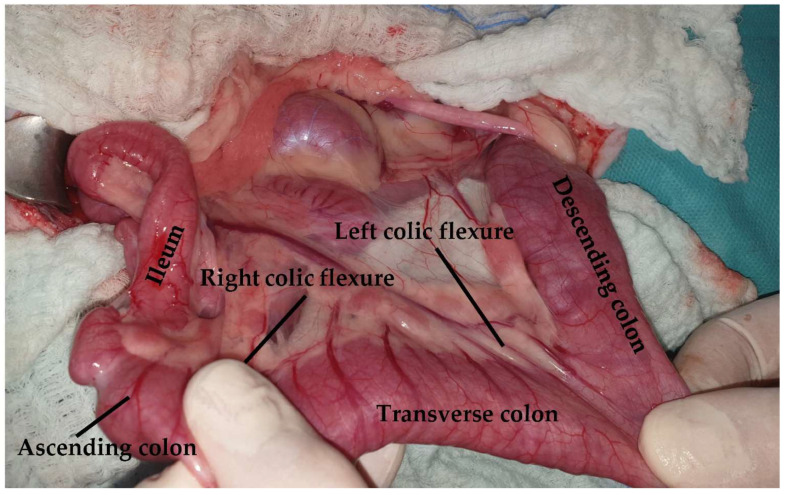
The colon is retracted to the right side of the abdomen to provide access to the left sublumbar region. The head is located on the left side of the image.

**Figure 18:**
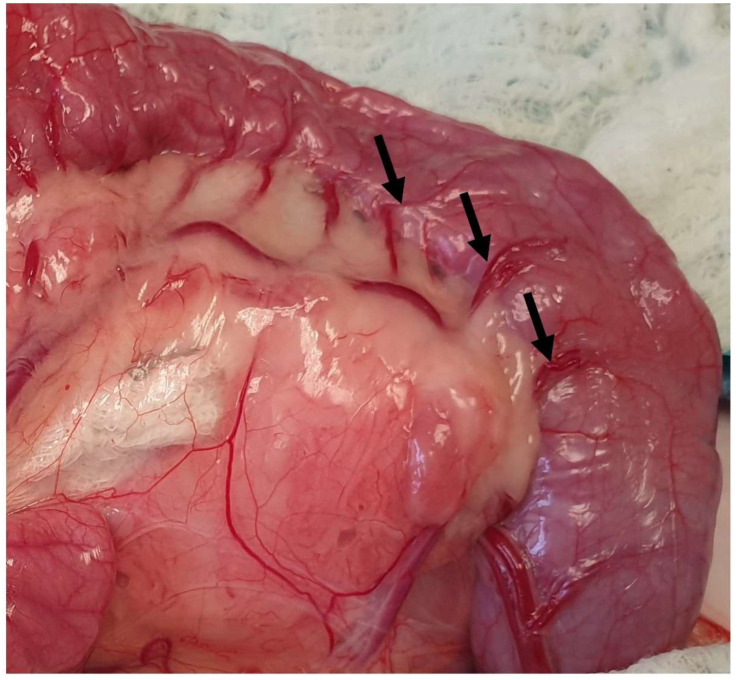
Vasa recta of the left colic and caudal mesenteric arteries are exposed (arrows). These vessels may be ligated during a subtotal colectomy.

**Figure 19:**
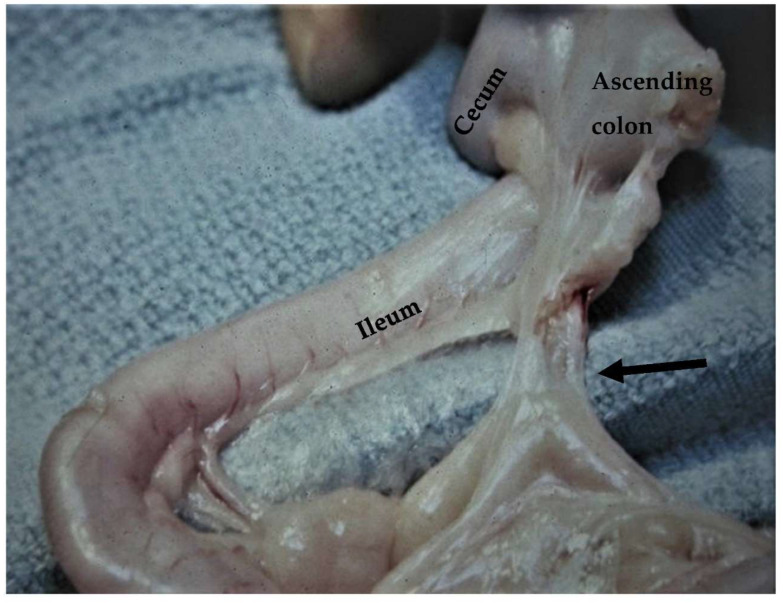
During subtotal colectomy with preservation of the ileocolic valve, the short mesentery supplying the ileocecocolic region (arrow) may contribute to increased tension at the anastomosis side (courtesy D.M. Bright).

The colon of the cat is subdivided into ascending, transverse, and descending colon (Figure 16). The ascending colon is located on the right side of the abdomen, extending from the cecocolic orifice to the transverse colon [20,89]. The right colic flexure connects these two parts of the large intestine [89]. Mesocolon and mesoduodenum stabilize the ascending colon [89]. The left colic flexure connects the transverse colon, which lies at the origin of the cranial mesenteric artery, along with the descending colon [20]. In cats, the identification of the different parts of the colon and the flexures connecting these parts is not feasible [20]. The descending colon, which is the longest part of the colon, extends up to the pelvic inlet [20]. The duodenocolic fold connects the ascending duodenum to the descending colon and can be transected to improve visualization and manipulation of the duodenum. During exploratory celiotomy, the descending colon is retracted to the right side of the abdomen in order to gain access to the left sublumbar region [20,89] (Figure 17). The descending colon is the anatomical region, in which a colopexy is performed either using an appositional or incisional technique [89,96]. Colopexy is indicated for the management of recurrent rectal prolapse. In both techniques, the colon is fixated in the left abdominal wall. In the incisional technique, as long as in the colon, the submucosa layer is thin, care must be taken not to penetrate the lumen. Laparoscopic-assisted colopexy with good long-term results was reported in a cat [97]. The ascending colon is supplied by the ileocolic artery, which is a branch of the cranial mesenteric artery. The ileocolic artery is subdivided into the right colic artery, which also supplies the ascending colon. The transverse colon is supplied by the middle and right colic branches of the ileocolic artery. The descending colon is supplied by the middle colic and left colic artery, which is a branch of the caudal mesenteric artery [89] (Figure 16). Subtotal colectomy is a commonly performed procedure in cats used for the management of megacolon. During subtotal colectomy, the right, middle, and left colic arteries are ligated and transected [98]. It is important during subtotal colectomy to preserve the caudal mesenteric artery and vein in order not to affect the blood supply of the remaining colon/rectum, even though there is little evidence that the resection of the caudal mesenteric artery affects the blood supply of the remaining colon and rectum [99,100]. Another option is the ligation of the vasa recta of the left colic and caudal mesenteric arteries to preserve the blood supply of the distal colon [101] (Figure 18). Subtotal colectomy with preservation of the ileocolic junction is the preferred technique, as removal of the ileocolic junction is associated with the increased frequency and severity of diarrhea [8]. Anastomosis should be performed under minimal tension. However, if the short mesentery supplying the ileocecocolic region contributes to increased tension at the anastomosis site, resection of the ileocolic junction is advised [100] (Figure 19). A surgical procedure rarely performed in the feline colon is end-on colostomy, which is usually used for the management of atresia ani and rectocutaneous or rectovaginal fistulas [102,103,104]. 

## 4. Digestive Glands

### 4.1. Liver

**Figure 20:**
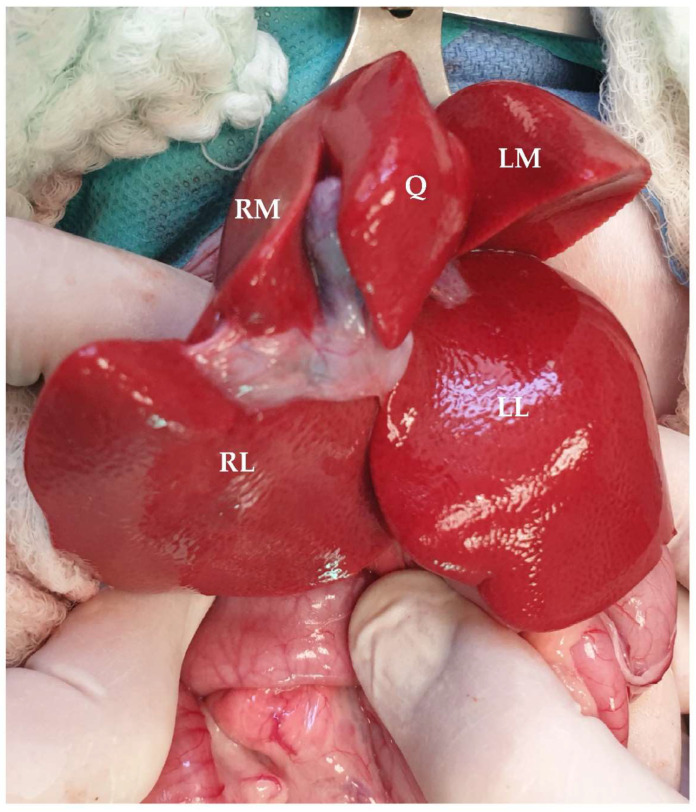
Visceral surface of the feline liver. (RM: right medial lobe; RL: right lateral lobe; Q: quadrate lobe; LM: left medial lobe; LL: left lateral lobe). Head is located in the upper part of the image.

The liver, which is located in the cranial abdomen and within the rib cage, is the largest gland of the gastrointestinal tract, representing 2% of the body weight of the cat [68,105,106]. The liver of the cat has a parietal surface in contact with the diaphragm and a visceral surface in contact with the stomach, duodenum, and right kidney [107]. The largest proportion of the liver lies on the right side [108]. The liver of the cat is further divided into 6 lobes: the left lateral and medial, the right lateral and medial, the quadrate, and the caudate lobe, which is further subdivided into the caudate and papillary processes [68]. The left lobe is divided into right and left sides via a cleft, making resection of these lobes less challenging compared to the right lobes [68]. The right lobes are also attached to the caudal vena cava, making dissection more challenging [109]. On the ventral parts of the left medial, quadrate, and caudate process of the caudate lobe, there are notches that offer a convenient site for liver biopsies [20] (Figure 20). 

**Figure 21:**
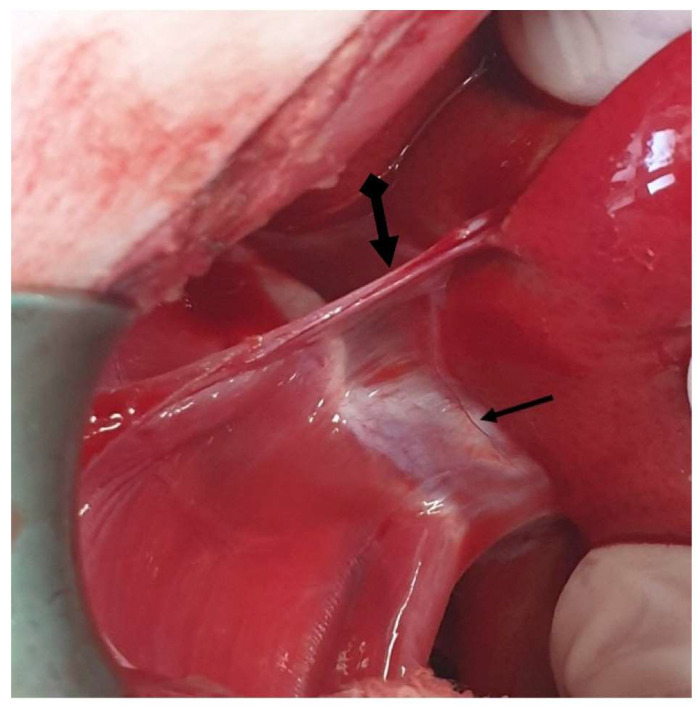
The liver is attached to the diaphragm via the coronary ligaments (pointed arrow). The caudal vena cava (pre-hepatic part) is also visualized (black arrow). The head is located on the left side of the image.

**Figure 22:**
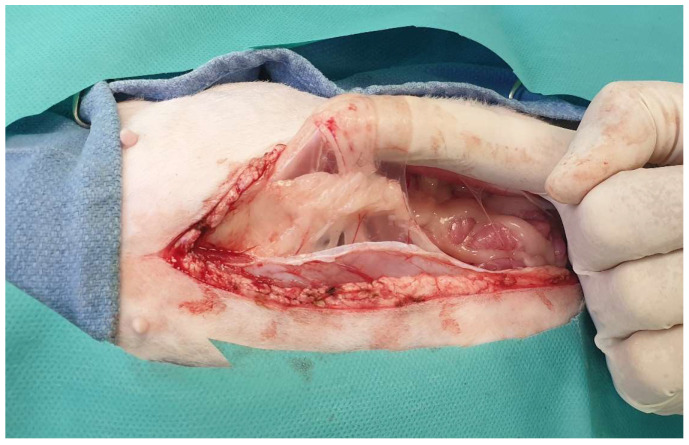
The falciform ligament is lifted using the surgeon’s index finger. The head is located on the left side of the image.

**Figure 23:**
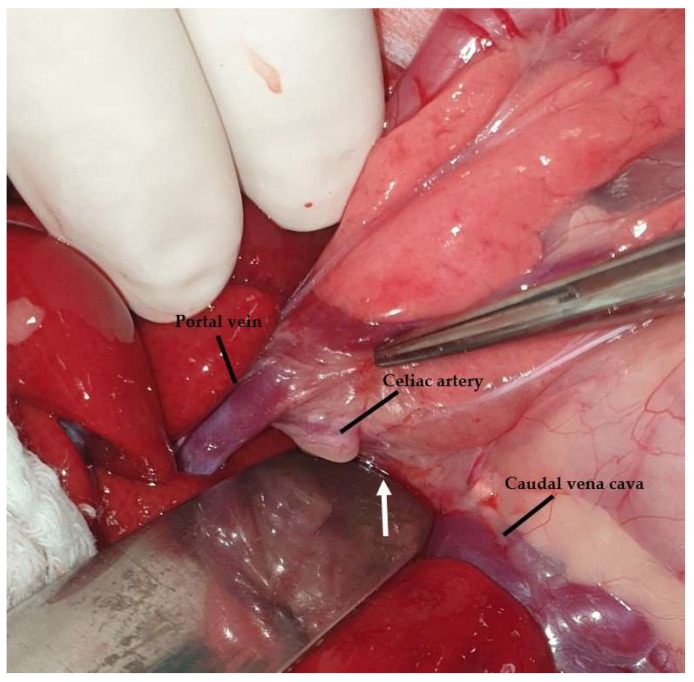
The epiploic foramen (a malleable retractor is inserted into the foramen [white arrow]) is bordered by the caudal vena cava dorsally and the portal vein ventrally. The celiac artery is also visualized. The head is located on the left side of the image.

**Figure 24:**
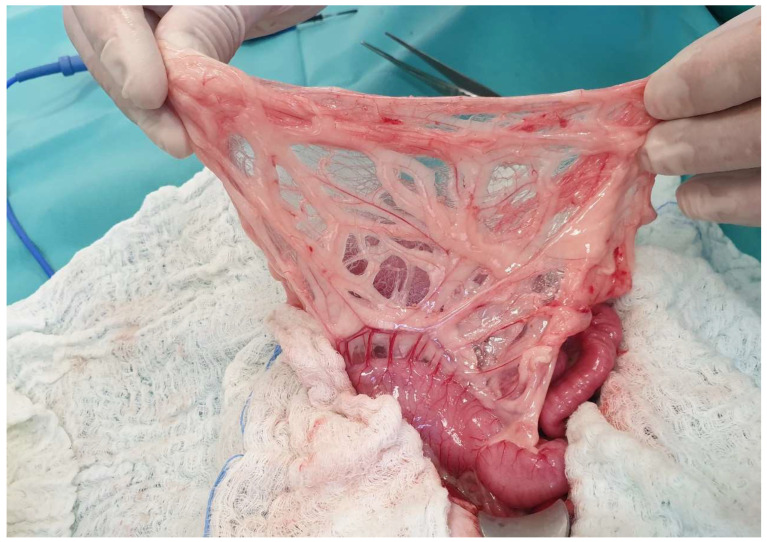
The omental bursa can be seen by ventrally lifting the greater omentum. The head is located in the upper part of the image.

**Figure 25:**
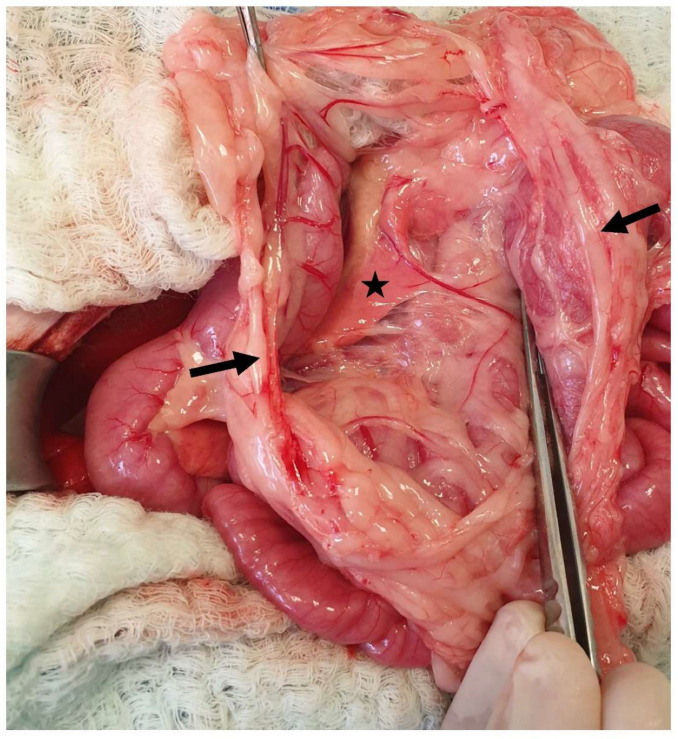
A hole (black arrows) was created in the ventral leaf of the greater omentum to gain access to the omental bursa. The left pancreatic lobe is lying within the bursa (asterisk). The head is located on the left side of the image.

The liver is attached to the surrounding anatomical regions via ligaments, including the coronary ligament, which connects the liver to the diaphragm, the two right triangular ligaments attached to the right lateral and medial lobes, the left triangular ligament attached to the left lobe, the hepatorenal ligament that connects the caudate lobe to the right kidney, and the hepatoduodenal and hepatogastric ligaments [68,105] (Figure 21). The triangular and coronary ligaments need to be transected for partial or total lobectomies to provide better exposure to the affected lobes [109]. The left triangular is also transected to determine exposure to portosystemic shunts (PSS) (left gastrophrenic, gastroazygos shunt) [31]. The hepatoduodenal and hepatogastric ligaments represent the lesser omentum that has previously been described [104]. During the lobectomy of the left lateral lobe, a window is created in the hepatoduodenal ligament to isolate and ligate the lobar hepatic artery, as described in dogs [110]. The lesser omentum may also need to be opened to perform gastrophrenic shunt isolation [31]. The falciform is the ligament extending from the umbilicus to the diaphragm (Figure 22). This ligament is usually transected during exploratory celiotomy in order to improve the visualization of the cranial abdomen [111]. Another clinically and surgically important anatomical region is the epiploic foramen, which is also known as foramen of Winslow, i.e., an opening into the omental bursa that is bordered by the caudal vena cava dorsally and the portal vein ventrally and located medial to the caudate lobe [68] (Figure 23). This knowledge of the anatomical side of the epiploic foramen is useful during celiotomies, especially during lobe lobectomies or cases of hemoperitoneum. More specifically, the access of epiploic foramen allows the Pringle maneuver technique, which occludes hepatic inflow. This maneuver can be used in liver lobectomies, the resection of large abdominal tumors (e.g., adrenal masses), or cases of severe cases of hemoperitoneum that require a temporary occlusion to allow the localization of the hemorrhage side [112,113]. The epiploic foramen is also the region in which some of the PSS may be found, including the splenocaval and the right gastrocaval shunt [31]. The other types of shunts, including the left gastrophrenic, the left gastroazygous, the left colocaval, and the left coloiliac shunts, are found at the level of the esophageal hiatus, aortic hiatus, and sixth or seventh thoracic vertebra, respectively [31]. The portoazygos shunt is found within the omental bursa, which needs to be opened by creating a hole in the ventral leaf of the greater omentum [114] (Figure 24 and Figure 25). More specifically, portocaval shunts are identified and attenuated at the epiploic foramen, just before their entrance to the caudal vena cava. Shunts entering the left phrenic vein are attenuated on the abdominal surface of the diaphragm. Right divisional intrahepatic shunts are usually pre-hepatically attenuated around the right branch of the portal vein or via intrahepatic dissection. Left divisional intrahepatic shunts are usually post-hepatically attenuated just before their entrance to the left hepatic vein or around the left hepatic vein. Central divisional intrahepatic shunts are usually attenuated pre-hepatically or intra-hepatically or via interlobar dissection. Μethods described for shunt attenuation in cats include ameroid constrictor, cellophane banding, and suture ligation, even though ligation is not considered to be a well-tolerated method [115,116]. A percutaneous transvenous coil embolization has also been used for intrahepatic PSS occlusion [117,118]. However, further investigation is needed, especially in cats, due to the small size of this species, which could potentially limit the use of endovascular techniques. 

**Figure 26:**
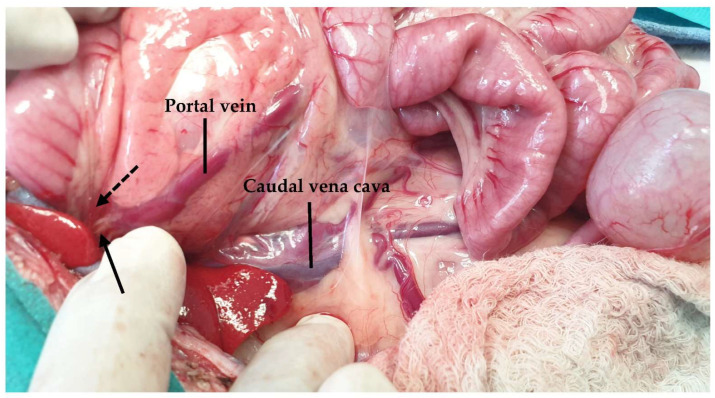
The portal vein (left branch: dotted arrow; right branch: black arrow) is exposed by ventrally and medially lifting the duodenum.

The blood supply in the feline liver is provided by the hepatic artery, which is a branch of the celiac artery, and the portal vein, which provides 80% of the blood supply to the liver [20,68]. Recently, Metzger et al. described the vascular anatomy of the liver in seven healthy cats [119]. Based on their results, it was reported that the portal vein, upon entering the liver hilus, is divided into a right and left branch, giving branches to the liver lobes, in contrast to previous reports for three branches [68] (Figure 26). When performing a liver lobectomy, care must be taken to preserve these branches of the portal vein [109]. In Metzger et al.’s study, it was found that the portal vein supplying the quadrate lobe came from one of the branches of the right medial lobe in four of the seven cats, a finding that has not been described in dogs. Further unique information compared to dogs was the presence of smaller portal veins that directly originated from the intra-hepatic main portal vein in two cats. In these seven cats, there were not many anatomical variations, even though the limitations in the number and breed of the cats could have affected the results [119]. The venous drainage of the feline liver is provided by the hepatic vein, which is divided into three major branches: the right, the central, and the left branches [119]. The fact that the right lateral lobe and caudate process of the caudate lobe shared a common hepatic primary venous branch in three liver specimens is important, especially during lobectomies of the left medial, quadrate, right medial lobe, and caudate processes of the caudate lobe [119]. All of this information is important to the surgical planning of feline liver lobectomies and supports using a pre-surgical CT examination to detect anatomical variations or anomalies.

The main surgical procedures performed on the feline liver include liver biopsy and partial or total liver lobectomy for the management of liver masses, lesions, or severe trauma. Liver biopsy in cats can be performed in different ways, including ultrasound-guided biopsies using automated devices, laparoscopic techniques with 5-mm forceps, or open surgical intervention [120,121]. However, in cats, it has been found that ultrasound-guided liver biopsies, which are performed using a Tru-Cut needle (usually 16 mm in length), have a risk of hemorrhage and should be avoided, especially in anemic patients [122,123]. For open surgical intervention, a new technique that has been described is the use of a pre-tied ligating loop for liver biopsy [124]. The same technique can be used to perform liver lobectomy. Liver lobectomy can be performed via hand suturing or the separation and ligation of the vessels in the hilus. The liver lobectomies in cats can also be performed using staplers. It has been proved that a TA stapler is effective for performing liver lobe resection in cats (usually the 2.5-mm or 3.5-mm cartridge stapler size) [125]. The use of an encircling suture for lobectomy should be avoided due to the risk of hemorrhage [107]. In general, during liver surgical procedures, the surgeon should use alternative methods of hemostasis to augment the staple line [125]. In cats, two case reports of transcatheter arterial embolization—one for the treatment of hepatocellular carcinoma and the other for initial hemostasis due to liver tumor hemorrhage—have been published [126,127]. Transcatheter arterial embolization needs further investigation in small animals, especially cats. 

**Figure 27:**
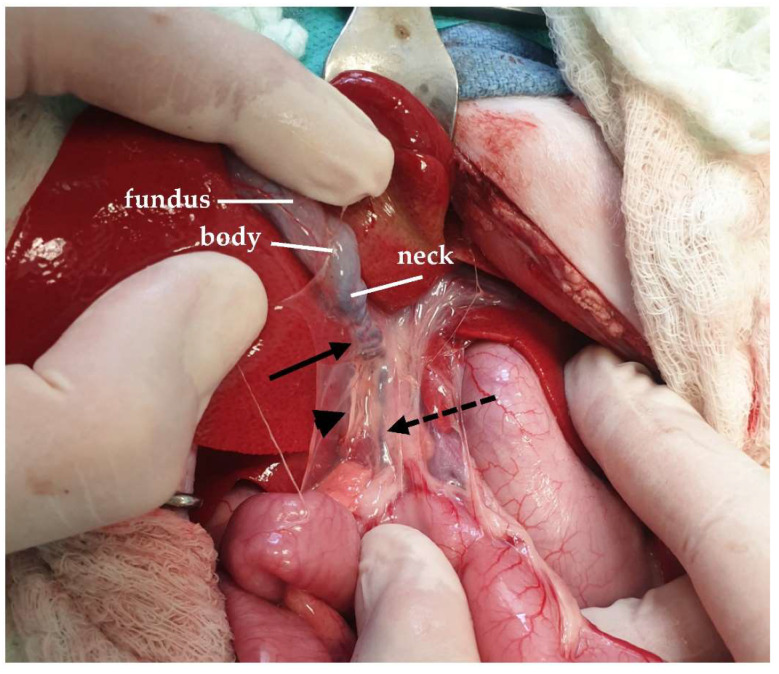
The gallbladder is exposed by lifting the quadrate lobe of the liver (black arrow: cystic duct; dotted arrow: common bile duct; arrowhead: hepatoduodenal ligament).

The gallbladder of the cat is located between the right medial and the quadrate lobe between the eighth and tenth intercostal spaces [128,129]. It consists of the fundus, the body, and the neck, with the fundus being the most caudal part [19,107] (Figure 27). There are differences described in the size, shape, and position of the gallbladder in cats [130]. An anatomical variation that is rarely described in cats is the duplication of the gallbladder [131,132,133]. The biliary system begins at the cystic duct, which is about 3 cm long and tortuous in cats, and extends from the neck of the gallbladder to the common bile duct. The common bile duct is the point at which the first hepatic duct, which collects the bile from the liver lobes, is joined to the cystic duct [68,128]. The common bile duct is formed either by the cystic and the left and right hepatic ducts or, rarely, by the cystic and one hepatic duct [134]. In cats, there is no common hepatic duct [20]. The long common bile duct in cats enters the duodenum at the major duodenal papilla, which is 3 cm caudal to the pylorus [68]. In total, 20% of the cats studied had an accessory pancreatic duct that entered the duodenum at the minor duodenal papilla 2 cm distal to the major papilla [68]. The major duodenal papilla can be approached through a duodenotomy for choledochal stent placement [72,135]. Sphincteroctomy can also be performed following an incision in the papilla [135,136]. The difference between cats and dogs is that, in cats, the common bile duct is conjoined with the pancreatic duct before enters the duodenum via the ampulla of Vater [70]. This connection between the common bile and pancreatic duct in cats is one of the explanations for the triaditis in cats, which refers to concurrent pancreatitis, cholangitis, and inflammatory bowel disease [137]. The biliary tree may need to be catheterized to investigate its patency in a normograde or retrograde manner. However, normograde catheterization is more challenging due to the angle that is formed between the cystic and common bile ducts [138]. The gallbladder is supplied by the cystic artery, which is a branch of the hepatic artery [107]. The cystic duct, along with its respective artery, is ligated during cholecystectomy [68]. The main surgical procedures that are performed in the biliary tree include cholecystostomy tube placement, choledochal stenting, cholecystectomy, cholecystotomy, choledochoduodenostomy, and choledochotomy [135]. A cholecystotomy is rarely performed in the apex of the gallbladder [68]. During cholecystoenterostomy, as well as either cholecystoduodenostomy or cholecystejejunostomy, the cystic artery must be preserved, and the cystic duct must remain intact and not become twisted [68,139,140,141]. Finally, choledochotomy is a procedure usually avoided due to the friability of the common bile duct and can only be performed in a dilated duct, and only a few cases have been described in cats [135,142,143]. 

### 4.2. Pancreas 

The pancreas of the cat, which consists of two lobes, i.e., the right and the left lobe, which fuse to form the body, has a length of about 12 cm and is a V-shaped organ that lies just caudal to the hepatic hilus [128,144,145]. The right lobe of the pancreas, which is short and has a hook-like shape, is closely related to the duodenum and located in the mesoduodenum [146]. The access of the right lobe is achieved by retracting the descending duodenum [146]. The left lobe, which initially follows the greater curvature of the stomach, lies within the dorsal leaf of the greater omentum [20,146]. Access to the left lobe is achieved via ventral retraction of the stomach and omentum or by opening the omental bursa. In the feline pancreas, there is only one pancreatic duct, which has a mean diameter of 0.8 mm, which is formed via the connection of two smaller ducts of the right and left lobe, entering the major duodenal papilla [145,147,148]. However, an accessory pancreatic duct has been described in 20% of cats opening 10 mm distal to the minor duodenal papilla [144]. The splenic artery supplies the left lobe of the pancreas, the cranial pancreaticoduodenal, which is a branch of the hepatic artery, supplies the proximal portion of the right lobe, and the right caudal pancreaticoduodenal supplies the distal part of the right limb [144,146]. Partial pancreatectomy is performed in cats for the management of abscesses, pseudocysts, and tumors. During pancreatectomy of the left limb, the branches derived from the splenic artery need to be ligated [144]. The distal part of the right limb is supplied by the caudal pancreatoduodenal branch of the cranial mesenteric artery. The pancreaticoduodenal vessels must be preserved during pancreatectomy to avoid duodenal necrosis [146]. Partial pancreatectomy can be performed using bipolar sealing devices [149]. Other surgical procedures in the pancreas include biopsies, in which the distal part of the right limb is preferred to avoid the pancreatic ducts [146]. Laparoscopic pancreatic biopsies can also be safely performed in cats using 5-mm biopsy punch forceps [150]. A total pancreatectomy has not been performed in cats. Cruciani et al. in 2022 described, for the first time, a pancreaticoduodenectomy performed on a cat for the removal of the left pancreatic limb via ligation of the pancreatic ducts [151]. The technique was performed with a vessel-sealing device and a suture fracture technique, and, despite the good results, further investigation is required to determine the long-term results [151].

## 5. Conclusions

In the present study, a detailed anatomy of the feline gastrointestinal tract was described, focusing on anatomical regions of surgical importance. Gastrointestinal surgery is commonly performed in cats. Thorough knowledge of the surgical anatomy of the feline gastrointestinal tract is essential to achieving successful outcomes and the avoidance of post-operative complications. 

## Data Availability

Not applicable.
